# Identification of novel and candidate miRNAs in rice by high throughput sequencing

**DOI:** 10.1186/1471-2229-8-25

**Published:** 2008-02-29

**Authors:** Ramanjulu Sunkar, Xuefeng Zhou, Yun Zheng, Weixiong Zhang, Jian-Kang Zhu

**Affiliations:** 1 Department of Biochemistry and Molecular Biology, Oklahoma State University, Stillwater, OK 74078, USA; 2 Department of Computer Science and Engineering, Washington University in St. Louis, 1 Brookings Drive, St Louis MO 63130, USA; 3 Department of Botany and Plant Sciences, University of California, Riverside, CA 92521, USA

## Abstract

**Background:**

Small RNA-guided gene silencing at the transcriptional and post-transcriptional levels has emerged as an important mode of gene regulation in plants and animals. Thus far, conventional sequencing of small RNA libraries from rice led to the identification of most of the conserved miRNAs. Deep sequencing of small RNA libraries is an effective approach to uncover rare and lineage- and/or species-specific microRNAs (miRNAs) in any organism.

**Results:**

In order to identify new miRNAs and possibly abiotic-stress regulated small RNAs in rice, three small RNA libraries were constructed from control rice seedlings and seedlings exposed to drought or salt stress, and then subjected to pyrosequencing. A total of 58,781, 43,003 and 80,990 unique genome-matching small RNAs were obtained from the control, drought and salt stress libraries, respectively. Sequence analysis confirmed the expression of most of the conserved miRNAs in rice. Importantly, 23 new miRNAs mostly each derived from a unique locus in rice genome were identified. Six of the new miRNAs are conserved in other monocots. Additionally, we identified 40 candidate miRNAs. Allowing not more than 3 mis-matches between a miRNA and its target mRNA, we predicted 20 targets for 9 of the new miRNAs.

**Conclusion:**

Deep sequencing proved to be an effective strategy that allowed the discovery of 23 low-abundance new miRNAs and 40 candidate miRNAs in rice.

## Background

In plants, transcriptional or post-transcriptional gene silencing is directed by genome-encoded 21–24-nt small RNAs. These small RNAs can be divided into two major classes: microRNAs (miRNAs) and short-interfering RNAs (siRNAs). miRNAs function in post-transcriptional gene silencing by guiding mRNA degradation or translational repression [1-4]. Endogenous siRNAs are further classified into two major groups predominantly based on the size and mode of gene regulation. The 21-nt size class siRNAs represented by trans-acting siRNAs (tasiRNAs) and others guide the cleavage of the target mRNAs at the post-transcriptional level, whereas 24-nt size class siRNAs are implicated in DNA and histone modifications leading to transcriptional gene silencing [5,6].

MicroRNAs were first identified in *Caenorhabditis elegans *through genetic screens for aberrant development [7,8] and were later found in almost all multicellular eukaryotes examined. Mature miRNAs are single-stranded ~21 nt small RNAs which are generated from a single-stranded primary transcript (pri-miRNA) forming an imperfect hairpin-like structure, by a series of enzymatic activities of double-stranded RNA recognizing RNase III enzymes (Drosha and DCR1 in animals and DCL1 in plants). The plant nuclear localized Dicer-like 1 (DCL1) enzyme processes pri-miRNA into a mature miRNA [9,10]. In Arabidopsis, the HYPONASTIC LEAVES 1, another double stranded RNA binding protein, together with SERRATE (SE), a C_2_H_2 _Zinc finger protein, co-operatively assists DCL1 in releasing the miRNA and miRNA* from the fold-back structure [11-14]. The released 21-nt duplex is stabilized by the addition of methyl groups to the 3'ends of the duplex and this step is catalyzed by the methyltransferase HEN1 [15]. The methylated miRNA-miRNA* duplex is exported to the cytoplasm by HASTY, the plant ortholog of exportin 5 and other yet unidentified exporters [3,16]. Subsequently, the duplex is unwound and only the active strand which is referred to as miRNA is loaded onto the RNA-induced silencing complex (RISC). Guided by miRNAs, the RISC recognizes the complementary sites on the target mRNAs to cause transcript cleavage [1,3,17] or translational arrest [2,4].

In plants, ~20 miRNA families which are well conserved between dicots and monocots are known. Of these, 7 miRNA families, i.e., miR156/157, miR160, miR159, miR319, miR165/166, miR390 and miR408 have been also found in primitive land plants such as *Physcometrella *and *Selaginella *suggesting that these are deeply conserved [18-21]. In addition, *Arabidopsis*, rice, *Populus *and *Physcometrella *possess many non-conserved lineage- or plant species-specific miRNA families [19,22-26].

Recent advances in high-throughput sequencing methods have revolutionized the identification of low-abundance non-conserved miRNAs in *Arabidopsis *[22,24-26]. The application of sequencing technologies such as MPSS and 454 resulted in finding 13 new miRNAs in Arabidopsis [24]. Similarly, with enhanced sequencing depth 38 new miRNAs were discovered in *Arabidopsis *[25]. Using a similar method coupled with the computational approach, 48 new miRNAs were identified in *Arabidopsis *[26]. Most of these newly identified miRNAs are not conserved in rice or *Populus *suggesting that these may be specific to *Arabidopsis*. Non-conserved miRNAs are thought to be the miRNAs which are evolving or evolved recently and only some of them with functional significance will be retained and the remainder may be lost in a short time scale [26].

The identification of a near complete set of small RNAs in any organism is of fundamental importance to understanding small RNA-mediated gene regulations and the diversity of small RNAs. It lays the necessary foundation for unraveling the complex small RNA-mediated regulatory networks controlling development, nutrient responses and tolerance to biotic and abiotic stresses [27-29]. Rice (*Oryza sativa *L. is an obvious choice for high-throughput small RNA analysis, because of its worldwide agricultural importance, besides being a model monocot plant with a sequenced genome. The known genome coupled with high-throughput transcriptome (coding and non-coding RNA) analyses will significantly advance our ability to unravel the small RNA-guided circuitry in rice. Thus far, computational and low-depth conventional sequencing of rice small RNA libraries have been successful in identifying conserved miRNAs, in addition to a few monocot-specific and rice-specific miRNAs [30-37]. Two recent studies reported large scale sequencing of small RNAs in rice and some of these sequences likely new miRNAs [38,39], but their annotation as new miRNAs has not been confirmed. Here, we used deep sequencing of rice small RNAs to identify novel miRNAs and possibly stress-regulated miRNAs. Sequence analysis indicated that we have found 23 novel miRNAs as well as 40 candidate miRNAs in rice. Six of these new miRNAs (Osa-miR1436 and five newly found members that belong to Osa-miR444 family i.e., Osa-miR444c.1, c.2, d, e, f) appear to be conserved in other monocots. Northern analysis revealed that several of these new miRNAs and candidate miRNAs are abundantly expressed. We predicted 20 new targets for 9 of the newly found miRNAs in rice. Thus this study advances our understanding of miRNAs in rice by discovering many new miRNAs and their potential target genes.

## Results

### miRNA expression profiling in rice seedlings

In the present study, we carried out high-throughput sequencing of small RNA libraries in order to identify low-abundance candidate new miRNAs and potential stress-responsive miRNAs in rice. Four-week-old rice seedlings were either untreated (served as control) or treated with 150 mM NaCl for 24 hrs (salt stress) or were dehydrated for 12 h (drought stress). A small RNA library was generated from each of the samples and sequenced at 454 life sciences. The obtained raw sequence reads (a total of 714,202 from 3 independent libraries) were processed computationally to remove the 5' and 3' adapter sequences and this yielded a total of 331,422 genome matching reads from 3 libraries (102,876, 54,016 and 174,530 reads from untreated, drought and salt libraries, respectively). The remaining sequences that could not mapped to the rice genome were discarded and possibly these sequences might contain sequence errors, affected by RNA editing, or be derived from the unsequenced genomic regions or contaminants. After removing rRNA, tRNA and sn/snoRNA, we obtained 58,781, 43,003 and 80,990 unique small RNAs that match perfectly with the rice genome from control, salt and drought libraries, respectively.

Currently, miRBase lists 243 rice miRNAs (miRBase release 10.1) that can be grouped into 62 miRNA families. Out of these, 9 miRNA families (miR413, miR414, miR415, miR416, miR417, miR418, miR419, miR420 and miR426) were only predicted based on sequence conservation between rice and Arabidopsis [33]. Thus far, these sequences were neither found in any of the deeply sequenced Arabidopsis small RNA libraries [24-26] nor in rice ([32,34-37], this study) thus, as of today the count of expressed miRNA families are set at 53 in rice. Several of them are non-conserved rice-specific miRNA families and their characterization as miRNAs was solely based on predicted fold-back structures for flanking sequences of cloned sequences [32,34-37]. Neverthelesss, the sequencing depth obtained in this study is sufficient to find 46 out of 53 expressed miRNA families known in rice. Previously reported seven miRNA families are not represented in our sequences, possibly due to their extreme low abundance or tissue- or cell-specific expression patterns or other unknown reasons such as biased cloning.

### New miRNAs

Defining new miRNAs in rice is particularly challenging since a rice *dcl1 *knockout mutant is not available. Rice *dcl1 *knock-down plants have been reported [35], but the material was unavailable for our study. Thus, our miRNA classification in this study is based on the following criteria: 1) predicted fold-back structures for the precursors, 2) in several cases, occurrence of the small RNA sequence in more than one independent library, 3) the presence of miR* sequence in atleast one of the libraries for several new miRNAs, and 4) their conservation in related monocots, if any. Most conserved miRNAs exists as gene families and are therefore represented by multiple loci in *Arabidopsis*, rice and *Populus *whereas recently evolved/evolving miRNAs are found to have a single locus in the genome [24-26]. In anology with these recent findings in *Arabidopsis*, we designated the small RNAs with a single locus in the rice genome as miRNAs. However, a few outliers with upto 3 loci in the rice genome were also classified as miRNAs in this study based on other considerations such as family members and their conservation in a related plant species. For instance, each of the three miR444 family members (miR444c.1, miR444c.2 and miR444e) can be mapped to more than one locus in the rice genome. Another miRNA, Osa-miR1436 with 20 hits in the rice genome has been considered as a miRNA because this appears to be conserved in *Aegilops*, a monocot. Together, we have designated 23 small RNAs obtained from 3 independent libraries as novel miRNAs in rice by applying the above criteria (Table 1). For all these 23 new miRNAs, fold-back structures could be predicted using the flanking genomic sequences to the cloned small RNAs (Additional file 1).

**Table 1:** Newly identified miRNAs in rice.

**miRNA_id**	**miRNA sequence**	**Length**	**Location**	**Number of loci in the rice genome**	**Frequency in the untreated library**	**Frequency in the drought-stressed library**	**Frequency in the salt-stressed library**
Osa-miR167a*	AUCAUGCAUGACAGCCUCAUUU	22	intergenic	1	0	0	1
Osa-miR810b.2	AAGUGAUUUAAUUAUGCCGUU	21	intergenic	1	8	4	0
Osa-miR444c.1	UGCAGUUGUUGUCUCAAGCUU	21	antisense to coding gene	3	3	0	1
Osa-miR444c.2	UGUUGUCUCAAGCUUGCUGCC	21	antisense to coding gene	2	3	2	4
Osa-miR444d	UUGUGGCUUUCUUGCAAGUUG	21	antisense to coding gene	1	1	0	0
Osa-miR444e	UGCAGUUGCUGCCUCAAGCUU	21	antisense to coding gene	2	2	0	1
Osa-miR444f	UGCAGUUGUUGCCUCAAGCUU	21	antisense to coding gene	1	3	0	1
Osa-miR1423	AGCGCCCAAGCGGUAGUUGUC	21	intergenic	1	2	0	1
Osa-miR1425	UAGGAUUCAAUCCUUGCUGCU	21	intergenic	1	8	0	5
Osa-miR1427	UGCGGAACCGUGCGGUGGCGC	21	intergenic	1	8	1	2
Osa-miR1428	CGUUUUGCAAAUUCGCAGGCC	21	intergenic	1	2	0	0
Osa-miR1429	GUUGCACGGGUUUGUAUGUUG	21	intergenic	1	1	0	0
Osa-miR1430	UGGUGAGCCUUCCUGGCUAAG	21	intergenic	1	5	2	4
Osa-miR1431	UUUGCGAGUUGGCCCGCUUGC	21	intergenic	1	4	3	
Osa-miR1432	AUCAGGAGAGAUGACACCGAC	21	intergenic	1	15	7	9
Osa-miR1435	UUUCUUAAGUCAAACUUUUU	20	intergenic	1	2	0	0
Osa-miR1436	ACAUUAUGGGACGGAGGGAGU	21	intergenic	20	77	24	0
Osa-miR1437	UCCGGCGCCGCACUAGGCACUG	22	intergenic	1	5	3	0
Osa-miR1438	AGGGUAAUUUUAUCAUUUUUA	22	intergenic	1	2	0	0
Osa-miR1439	UUUUGGAACGGAGUGAGUAUU	21	intergenic	1	0	0	2
Osa-miR1440	UGCUCAAAUACCACUCUCCU	20	intergenic	2	0	2	0
Osa-miR1441	ACCGGAUGUCGGAAAAGGUUU	21	intergenic	3	0	24	0
Osa-miR1442	AUUCAUAGUACUAGAUGUGU	20	intergenic	2	0	0	5

### Origin and frequency of the new miRNAs

**Figure 1:**
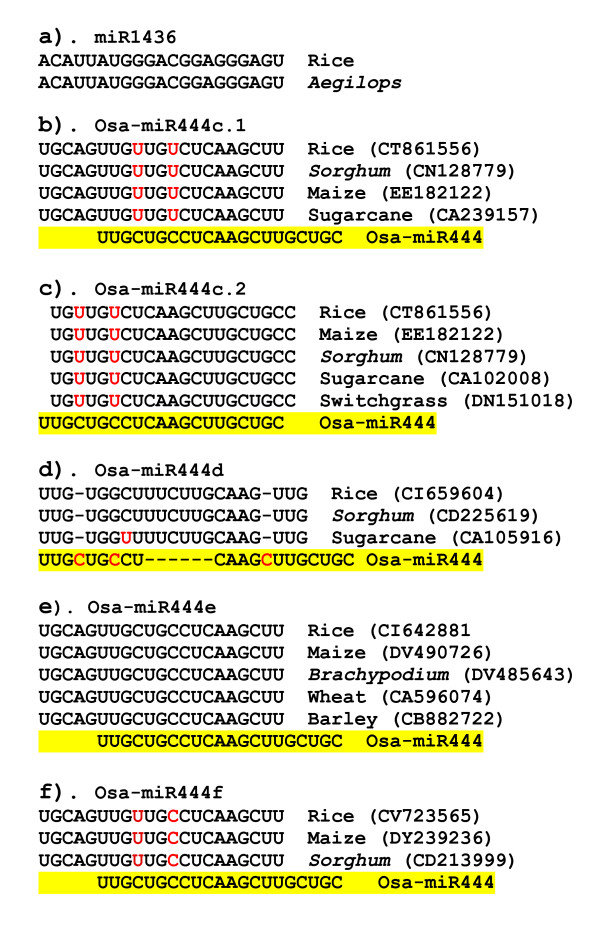
**miRNA sequence alignments of the new miRNAs conserved in monocots.** Figure 1a). Osa-miR1436 sequence conserved between rice and *Aegilops*. Figure b-f). miRNA sequence alignment of the newly identified rice miRNAs with the Osa-miR444. The Newly found miRNAs are conserved in related monocots and are included in the alignment. Osa-miR444 is highlighted with the yellow background. Red colored nucleotides in the newly identified miRNA in rice and other monocots indicate a nucleotide that is different from Osa-miR444.

In plants, most known miRNAs are found in between the coding genes, although a few are found in sense or antisense orientations in introns and exons of protein-coding genes. To identify the locations of precursors on the rice genome, we used the TIGR GenBank database. Of the 23 new miRNAs listed in the Table 1, most can be mapped to intergenic regions, although five new miRNAs (Osa-miR444c.1, c.2, d, e, f) belonging to Osa-miR444 family were found in the opposite strand of the protein coding genes. Sixteen of these new miRNAs can be mapped to single locus in rice genome (Table 1). Four miRNAs (Osa-miR444c.2, Osa-miR444e, Osa-miR1440 and Osa-miR1442) are mapped to 2 loci each, while 2 others (Osa-miR444c.1 and Osa-miR1441) are mapped to 3 loci each in the rice genome. Osa-miR1436 could be mapped to 20 loci (although hairpin structures could be predicted for the precursors from two loci only) and this has been included in the list of new miRNAs, because we found miR1436 homolog in *Aegilops*, a monocot (Figure 1a).

The miRNA frequencies ranged between 1 to 4948 reads in the control library. As expected, the top scoring sequences are conserved miRNAs. Compared to the conserved miRNAs, all of the new miRNAs were relatively low in abundance as indicated by their frequencies (Table 1). Four of the new miRNAs (Osa-miR1427, Osa-miR1430, Osa-miR1432 and Osa-miR444c.2) were found in all three libraries and 9 of the new miRNAs (miR444c.1, Osa-miR444e, Osa-miR444f, Osa-miR1423, Osa-miR1425, Osa-miR1431, Osa-miR1436, Osa-miR1437 and Osa-miR810b.2) appeared at least in 2 of the 3 libraries. Together, 13 of the new miRNAs appeared at least in more than one independent library. Three of the new miRNAs appeared only once (Osa-miR167*, Osa-miR444d and Osa-miR1429) in our sequences whereas the remaining 20 appeared multiple times. These findings suggest that a large number of miRNAs may be unique to the rice genome, and possibly many more low abundance miRNAs remain to be identified.

### Presence of miRNA*

The multitude of other endogenous small RNAs, some of which may originate from regions with a fortuitous potential to fold into miRNA-like hairpin structures, has complicated miRNA identification in plants, leading to the suggestion that biogenesis requirements be confirmed using mutants prior to annotation [28]. In the absence of expression analysis in *dcl1 *mutant, the detection of miRNA* sequences is an important criterion, because it supports the release of miRNA duplex from the predicted foldback structure [24-26]. At least 4 of our new miRNA sequences (Osa-miR1435, Osa-miR1430, Osa-miR1432 and Osa-miR1431) had their miR* sequences in our libraries.

### Some of the newly identified miRNAs are conserved in closely related monocots

**Figure 2:**
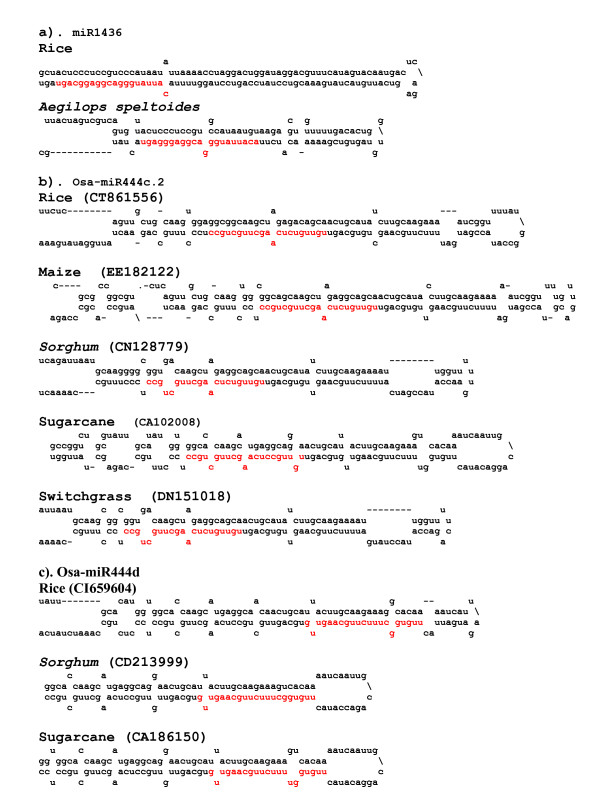
**Some of the newly identified miRNAs are conserved in monocots.** Predicted fold-back structure using the miRNA precursor sequences from rice and other monocots. Used EST sequences for prediction of fold-back structures are indicated in parentheses. Cloned miRNA sequence in the fold-back structures is shown in red letters.

To determine whether any of these novel candidate miRNAs are conserved among other plant species, we searched the nucleotide databases for homologs. This analysis indicated that 2 miRNAs, namely, Osa-miR1436 and five members of the Osa-miR444 family are conserved in several other monocots (Figure 1). Hairpin structures can be predicted for the miRNAs in these plant species using miRNA surrounding sequences obtained from EST databases (Figure 2). Previously, we have reported the identification of two miRNAs, miR436 and miR444 in rice [32]. In both cases, only the processed precursor transcripts can form hairpin structures but not using the genomic loci [32]. In this study, we have found 5 new members (Osa-miR444c.1, c.2, d, e, f) belonging to miR444 family (Figure 1). In all these cases, prediction of fold-back structures require the processed transcript but not for the genomic sequences surrounding the miRNA sequences in rice (Figure 2). Osa-miR444e and the previously reported miR444 sequences appears to be derived from one precursor with a shift of 5 nucleotides as indicated in Figure 1e. Interestingly, these 5 new members (Osa-miR444c.1, c.2, d, e, f) are highly conserved in several monocotyledonous plants such as *Sorghum*, maize, barley, wheat, switchgrass, sugarcane and *Brachypodium *(Figure 1b–f). It is worth mentioning here that although Osa-miR444d sequence appeared to be very different when aligned with the miR444 (Figure 1d) but both sequences show high complementarity with the one of the MADS box factors (Os02g49840). Thus, we consider that Osa-miR444d is another new member of miR444 family. Taken together, the absence of these miRNA sequences in *Arabidopsis *and their presence in closely related monocots suggest that these 6 miRNAs (Osa-miR1436 and Osa-miR444c.1, c.2, d, e, f) are specific to monocots.

### Predicted targets of the new miRNAs

The targets of conserved miRNAs can be predicted with very high confidence, whereas in single-genome analysis only the more extensively paired interactions can be predicted with reasonable confidence [30]. In order to reduce the ratio of false positive predictions we used a very stringent criterion for target predictions, i.e., simple blast searches for antisense hits with not more than 3 mismatches [40], although this method might miss some authentic targets. Furthermore, we focused on finding target sites in coding regions since previous studies found that plant miRNA target sites are predominantly located in ORFs [40]. This resulted in prediction of 20 targets for 9 of the new miRNAs (Table 2). Some of the interesting predicted target transcripts are MADS-box transcription factors, Zn-finger (C3HC4) family protein, EF-hand family proteins and a calmodulin-binding protein (proteins implicated in calcium signaling) and several pentatricopeptide repeat proteins. Thus, the non-conserved miRNA target genes encode a broad range of proteins (Table 2). It is interesting to note that target validations for non-conserved miRNAs in *Arabidopsis *had not been very successful, unlike conserved miRNA targets [26]. Future experimental validation will determine how many of these predicted targets are genuinely targeted by miRNAs in rice. For the remaining new miRNAs, we were unable to identify any targets using the above criterion. It has been hypothesized that "young" miRNAs, i.e. non-conserved and recently evolved miRNAs, exist without actual targets [26]. It is also possible that the applied stringent criteria might have missed the prediction of potential target genes.

**Table 2:** Predicted targets for the newly identified miRNAs in rice.

**miRNA**	**Target gene **(number of mismatches between the miRNA and the mRNA is indicated in parentheses)	**Putative function of the target gene**
Osa-miR1436	Os03g26190(3); Os06g23090(3); Os08g36050(1)	hypothetical proteins
Osa-miR1432	Os03g59770(1); Os03g59790(1); Os03g59870(1)	EF hand proteins
Osa-miR444e	Os04g38780(0); Os02g49840(2)	MADS-box factor 27; MADS-box factor 57
Osa-miR444c.1	Os02g49840(0); Os04g38780(2); Os08g06510(2)	MADS-box factor 57; MADS-box factor 27; zinc finger, C3HC4 type family protein
Osa-miR444f	Os04g38780(1); Os02g49840(1); Os08g06510(3)	MADS-box factor 27; MADS-box factor 57; zinc finger, C3HC4 type family protein
Osa-miR444c.2	Os04g38780(0); Os02g49840(2)	MADS-box factor 27; MADS-box factor 57
Osa-miR444d	Os02g49840(0); Os10g27170(3)	MADS-box factor 57; calmodulin binding protein
Osa-miR1427	Os04g32560(3); Os06g14810(3)	clpA homolog CD4B; 3-ketoacyl-CoA synthase
Osa-miR1425	Os10g35640(2); Os08g01640(3); Os08g01650(3); Os10g35230(3); Os10g35240(3); Os10g35436(3); Os08g01870(3); Os10g35790(3)	PPR proteins

### Putative new miRNAs

In addition to the new miRNAs listed in the Table 1, we have identified another 40 small RNAs as candidate miRNAs (Table 3). Thus far, miR395, a known conserved miRNA could be mapped to the 26 loci in rice (miRBase). The number of loci for each of these putative miRNAs is highly varied (4 to 306 loci) in the rice genome. In our list of 40 candidate miRNAs, one of them (cpmiR188) can be mapped to as many as 306 loci in rice. We were able to detect 7 of the candidate miRNAs (spmiR63, spmiR37, dpmiR4, cpmiR7, dpmiR26, cpmiR182 and cpmiR188) using small RNA blot analysis out of 12 tested. Some of these sequences with a very high number of hits to the rice genome could be siRNAs derived from repeat-rich regions that happen to have predicted fold-back structures. Fold-back structures can be predicted for at least one-third of these loci for each of the putative miRNAs. The fact that fold-back structures can be predicted for many loci suggests that some of these could be authentic loci for the miRNA. Regardless of whether or not these are derived from fold-back structures or dsRNAs, some of these small RNAs appeared several times in our libraries and some others with maximum number of loci (cpmiR182 with 212 loci and cpmiR188 with 306 loci) could be detected using small RNA blot analysis, suggesting that these are processed and accumulate at detectable levels. Future studies using rice *dcl *mutants will help resolve how many of these will be true miRNAs. We also predicted 22 targets for 10 of the candidate miRNAs in rice (Additional file 2).

**Table 3:** Newly identified candidate miRNAs in rice.

**Putative miRNA_id**	**Putative miRNA sequence**	**Length**	**Location**	**Number of hits to rice genome**	**Frequency in the untreated library**	**Frequency in the drought-stressed library**	**Frequency in the salt-stressed library**
spmiR56	UCUCUUAUAUUUUGAGUGGUCA	22	intergenic	1	0	0	1
spmiR58	UGGAUGGACCUGGAGCAUCGAC	21	intergenic	1	0	0	2
cpmiR37	CGCUAAUGUUGCAGCAAACUG	20	intergenic	2	2	0	0
dpmiR41	UACCCGGUUUUGCAGUCAAGGG	22	intergenic	4	0	11	0
spmiR63	UUUGGACGGAGGGAGUAUCUC	21	intergenic	4	0	0	7
cpmiR99	CGAGUCAUGCAACCAAUCACUG	22	intergenic	5	3	0	2
cpmiR130	ACGCUAUUGUUGCAGCAAACUG	21	intergenic	6	2	0	2
cpmiR34	UUUGAGACGGAGGGAGUAACU	21	intergenic	7	31	0	0
cpmiR164	UUCGAUAGGUACCUUGUCGA	20	intergenic	7	1	5	0
cpmiR96	GGAUGUCGGAAGAGGUUUUUA	20	intergenic	8	11	0	0
cpmiR5	AGAAUGUGUCACAUCCGGUACU	22	intergenic	9	15	0	24
cpmiR123	CCACGUGUAAAGAAGACCCGGU	22	intergenic	9	3	4	2
cpmiR179	UAUUAGGAUGUGUUACAUCC	20	intergenic	9	17	0	0
spmiR37	UACAUUUUAGGACGGAGGAA	20	intergenic	9	0	2	9
cpmiR19	GAUCCUUGAGGGCUAAUUCA	20	intergenic	10	4	2	0
dpmiR50	ACGAUCAAACGUUGGGCACG	20	intergenic	11	0	12	0
cpmiR148	GCCAAAUCAGAUGGAGAGUU	20	intergenic	13	6	2	0
cpmiR79	UCUAGUACUAUGAAUCUGGAU	21	intergenic	15	5	0	4
cpmiR155	GCGCACGGAGGUGAGGAACC	20	intergenic	15	14	7	0
cpmiR185	CCAACUUUGAUCGUCCGUUUU	21	intergenic	15	12	0	7
spmiR44	UUUGUCGGAUGAAGAAAUGACU	22	intergenic	15	0	0	12
cpmiR175	AUGAGACGGAGGGAGUAUAU	20	intergenic	20	19	0	0
dpmiR4	ACUGUUUGACCACUCGUUUUA	21	intergenic	20	0	10	0
dpmiR10	UGUGGCAUGCCACACGGACAU	21	intergenic	20	0	10	0
dpmiR56	CGUUGUAUCUGGUUUUGCGGUU	22	intergenic	20	0	11	0
cpmiR47	UUCGUCCCUUGACCGCAAAAC	21	intergenic	22	3	3	0
dpmiR15	AUUUUGAGUUUUUGUUUGUAUU	22	intergenic	23	0	4	0
cpmiR3	UUAUGAGACGGAGGGAGUAC	20	intergenic	27	17	0	0
cpmiR7	AGACGAGUGGUCAAACAGUGU	21	intergenic	35	87	5	0
spmiR27	UGGCUAUAUUUAGUUUGCUG	20	intergenic	37	0	0	12
dpmiR26	UUUUUAUAGGACGGAGGGAGU	21	intergenic	39	0	11	0
cpmiR116	UUGCACUGUUUGACCAUUCGUC	22	intergenic	52	24	0	0
cpmiR105	CGAUUUUCGUCCUUCAACCG	20	intergenic	58	11	0	0
spmiR31	GGUGGCAGGAGGACGGCGCCA	21	intergenic	68	0	2	3
cpmiR74	UGACCCUAAACCACAAAACC	20	intergenic	72	10	0	0
cpmiR110	UAAGACGGACGAUCAAAGUUG	21	intergenic	76	13	0	6
cpmiR85	ACGAAUUACCCCCCUCGACC	20	intergenic	121	8	2	2
cpmiR32	UGCCCGUGCGUUGCAACGGGU	21	intergenic	129	13	0	9
cpmiR182	UGACUAUCAAAAGUAGAUGGAGG	23	intergenic	212	9	1	18
cpmiR188	ACUAUCAAAAGUAGAUGGAG	20	intergenic	306	11	1	0

### Expression profiles of conserved miRNA families in rice seedlings

Even at relatively low sampling depths, many conserved miRNAs were sequenced in rice small RNA libraries indicating their greater abundance [32]. In agreement with this, the conserved miRNAs make up the top 10 most abundant signatures in our sequence reads (Table 4).

**Table 4:** Expression of previously reported conserved and non-conserved miRNAs in rice.

**miRNA**	**miRNA sequence**	**Frequency in the untreated library**	**Frequency in the drought-stressed library**	**Frequency in the salt-stressed library**
Osa-miR156a-j	UGACAGAAGAGAGUGAGCAC	565	302	436
Osa-miR156k	UGACAGAAGAGAGAGAGCACA	9	0	9
Osa-miR156l	CGACAGAAGAGAGUGAGCAUA	520	271	390
Osa-miR159a, b	UUUGGAUUGAAGGGAGCUCUG	92	16	45
Osa-miR159c	AUUGGAUUGAAGGGAGCUCCA	89	15	41
Osa-miR159d	AUUGGAUUGAAGGGAGCUCCG	79	13	37
Osa-miR159f	CUUGGAUUGAAGGGAGCUCUA	90	16	41
Osa-miR159e	AUUGGAUUGAAGGGAGCUCCU	0	0	0
Osa-miR160a-d	UGCCUGGCUCCCUGUAUGCCA	71	43	71
Osa-miR160e	UGCCUGGCUCCCUGUAUGCCG	66	41	68
Osa-miR160f	UGCCUGGCUCCCUGAAUGCCA	30	15	21
Osa-miR162a	UCGAUAAACCUCUGCAUCCAG	6	0	1
Osa-miR162b	UCGAUAAGCCUCUGCAUCCAG	3	0	1
Osa-miR164a, b, f	UGGAGAAGCAGGGCACGUGCA	55	32	38
Osa-miR164c	UGGAGAAGCAGGGUACGUGCA	12	13	20
Osa-miR164d	UGGAGAAGCAGGGCACGUGCU	45	22	32
Osa-miR164e	UGGAGAAGCAGGGCACGUGAG	38	22	34
Osa-miR166a-d, f, n	UCGGACCAGGCUUCAUUCCCC	23	12	27
Osa-miR166e	UCGAACCAGGCUUCAUUCCCC	14	5	17
Osa-miR166g, h	UCGGACCAGGCUUCAUUCCUC	24	12	26
Osa-miR166i, j	UCGGAUCAGGCUUCAUUCCUC	1	0	0
Osa-miR166k, l	UCGGACCAGGCUUCAAUCCCU	5	6	4
Osa-miR166m	UCGGACCAGGCUUCAUUCCCU	24	13	27
Osa-miR167a-c	UGAAGCUGCCAGCAUGAUCUA	106	34	36
Osa-miR167d-j	UGAAGCUGCCAGCAUGAUCUG	111	34	36
Osa-miR168a	UCGCUUGGUGCAGAUCGGGAC	1007	533	388
Osa-miR169a	CAGCCAAGGAUGACUUGCCGA	916	230	283
Osa-miR169b, c	CAGCCAAGGAUGACUUGCCGG	916	230	285
Osa-miR169d	UAGCCAAGGAUGAAUUGCCGG	8	1	0
Osa-miR169e	UAGCCAAGGAUGACUUGCCGG	925	237	289
Osa-miR169f, g	UAGCCAAGGAUGACUUGCCUA	939	238	291
Osa-miR169h-m	UAGCCAAGGAUGACUUGCCUG	943	242	294
Osa-miR169n, o	UAGCCAAGAAUGACUUGCCUA	271	67	76
Osa-miR169p	UAGCCAAGGACAAACUUGCCGG	2	1	1
Osa-miR169q	UAGCCAAGGAGACUGCCCAUG	28	11	3
Osa-miR171a	UGAUUGAGCCGCGCCAAUAUC	16	4	7
Osa-miR171b-f	UGAUUGAGCCGUGCCAAUAUC	82	28	52
Osa-miR171g	GAGGUGAGCCGAGCCAAUAUC	1	1	0
Osa-miR171h	GUGAGCCGAACCAAUAUCACU	0	2	0
Osa-miR171i	GGAUUGAGCCGCGUCAAUAUC	8	0	2
Osa-miR172a, d	AGAAUCUUGAUGAUGCUGCAU	402	100	192
Osa-miR172b	GGAAUCUUGAUGAUGCUGCAU	377	94	179
Osa-miR172c	UGAAUCUUGAUGAUGCUGCAC	377	94	177
Osa-miR319a, b	UUGGACUGAAGGGUGCUCCC	23	3	5
Osa-miR390	AAGCUCAGGAGGGAUAGCGCC	55	30	33
Osa-miR393a-b	UCCAAAGGGAUCGCAUUGAUCU	123	23	47
Osa-miR394	UUGGCAUUCUGUCCACCUCC	1	1	2
Osa-miR395a-t	GUGAAGUGCUUGGGGGAACUC	1	0	2
Osa-miR395o	AUGAAGUGUUUGGAGGAACUC	1	0	0
Osa-miR396a-c	UUCCACAGCUUUCUUGAACUG	8	1	4
Osa-miR396d, e	UCCACAGGCUUUCUUGAACUG	20	5	8
Osa-miR397a, b	UUAUUGAGUGCAGCGUUGAUG	10	5	11
Osa-miR398a, b	UGUGUUCUCAGGUCGCCCCUG	21	5	3
Osa-miR399e-g	UGCCAAAGGAGAUUUGCCCAG	1	0	0
Osa-miR408	CUGCACUGCCUCUUCCCUGGC	0	0	1
Osa-miR435	UUAUCCGGUAUUGGAGUUGA	4	0	1
Osa-miR437	AAAGUUAGAGAAGUUUGACUU	4	2	0
Osa-miR439a-j	UGUCGAACCGCGGUUGUUCGA	4	1	0
Osa-miR440	AGUGUCUCCUGAUGAUCGGGACAA	4	1	1
Osa-miR441a-c	UACCAUCAAUAUAAAUGUGGGAAA	2	2	0
Osa-miR442	UGACGUGUAAAUUGCGAGACGAAU	6	0	0
Osa-miR444	UUGCUGCCUCAAGCUUGCUGC	3	0	1
Osa-miR445a-i	UAAAUUAGUGUAUAAACAUCCGAU	4	0	0
Osa-miR446	CAUCAAUAUGAAUAUGGGAAAUGG	2	2	3
Osa-miR528	UGGAAGGGGCAUGCAGAGGAG	0	0	1
Osa-miR529	CUGUACCCUCUCUCUUCUUC	1	1	1
Osa-miR530	AGGUGCAGAGGCAGAUGCAAC	22	6	1
Osa-miR531	CUCGCCGGGGCUGCGUGCCGCCAU	3	1	4
Osa-miR535	UGACAACGAGAGAGAGCACGC	10	7	36
Osa-miR806a-h	AUGUGCUAAAAAGUCAACGGUG	19	3	9
Osa-miR808	AUGAAUGUGGGAAAUGUAAGAA	3	1	0
Osa-miR809a-h	UGAAUGUGAGAAAUGUUAGAAU	7	4	1
Osa-miR811a-c	ACCGUUAGAUCGAGAAAUGGACGU	4	1	0
Osa-miR812a-e	GACGGACGGUUAAACGUUGGAC	3	1	2
Osa-miR813	GGGUUAUGGAAUGGGUUUUACC	3	3	1
Osa-miR814a-c	CACUUCAUAGUACAACGAAUCU	0	2	1
Osa-miR815a-c	AAGGGGAUUGAGGAGAUUGGG	2	2	0
Osa-miR816	GUGACAUAUUUUACUACAAC	0	0	0
Osa-miR817	UCCAACUUGAGGCCCGAUUGA	0	0	0
Osa-miR818a-e	AAUCCCUUAUAUUAUGGGACGG	21	5	3
Osa-miR819a-k	UCAGGUUAUAAGACUUUCUAGC	1	2	0
Osa-miR820a-c	CGGCCUCGUGGAUGGACCAGG	55	17	26

The obtained sequence count data for conserved miRNAs in each of the libraries suggested that the depth of sequencing could be sufficient to support estimation of the expression levels of conserved miRNAs between the conditions represented by the 3 libraries. Qualitatively, we did not find any striking differences for conserved miRNAs in terms of their presence or absence in the stress libraries, although some notable quantitative differences exist. The most abundant miRNA family across the three libraries was miR169 (Table 4). If the abundance of a single miRNA family is calculated relative to the total number of miRNAs, the miR169 family alone accounted for 43%, 37% and 38% of the total miRNAs in the control, drought and salt stress libraries, respectively. Similarly, 2 miRNA families i.e., miR168 and miR156 families each of them accounted for 15% of the total miRNAs in these 3 libraries.

Different members in a miRNA family may have diverged slightly from each other and may even target different sets of messenger RNAs [41]. It is thus important to determine which variant is more abundantly expressed in a given tissue to determine their potential for target regulation. In Arabidopsis, recently Xie et al. [42], have reported the expression of 73 MIRNA genes by sequencing several small RNA libraries and by mapping transcription start sites for several loci [42]. To examine which of the miRNA family members are more abundantly expressed in rice seedlings, we inspected the expression profile of known miRNAs in untreated seedlings (control library) based on their sequence frequencies. Our approach is sequence based determination of abundance which allows us to verify which miRNA locus within a miRNA family is expressed in a given tissue. However, this is only possible if the different members differ at least in one-nt and are each represented by a single locus in the genome. The most abundantly expressed miRNA family in rice seedlings is miR169 which is represented by 8 members corresponding to 17 loci in rice (Table 4). The expression of four members of the rice miR169 family has been reported previously [32]. Here, we found evidence for the expression of all 9 variants in rice and their frequencies were highly varied (2 to 943). Five variants (miR169a, d, e, p and q) are represented by single copy whereas the 4 other variants have multiple copies in the rice genome. Interestingly 5 members (miR169a; miR169b, c; miR169e; miR169f, g; miR169h-m) had similar frequencies (916, 925, 939 and 943), although, only miR169a and miR169e, are derived from a single locus whereas the other 3 members have multiple paralogous loci in the rice genome (Table 4).

miR156 is the second most highly expressed miRNA in rice seedlings as determined by the number of reads (Table 4). miR156 is represented by 3 members (miR156a-j; miR156k and miR156l) that slightly differ in their sequences. The 3 miR159 members are represented by 12 different loci in the rice genome, and all 3 members were found to be expressed. The two members corresponding to miR156a-j (paralogous loci yield similar mature sequences which makes it difficult to judge which locus is expressed) and miR156l loci are almost equally represented in our sequences (about 565 times), whereas the third member (156 k) is also expressed but at 50 times lower level compared to the other 2 variants.

Rice miR168 is represented by two members (miR168a and miR168b) that differ slightly in nucleotide sequence and arise from two separate loci. Since each locus is distinguishable because of the miRNA sequence variation it can be easily determined whether or not both loci are expressed in rice. Sequence analysis indicated that only miR168a was expressed and represented by 1007 reads in our library but none for miR168b (Table 4). These findings are consistent with previous small scale sequencing of rice small RNA libraries wherein the expression of miR168a but not miR168b was observed [32]. These results suggest that miR168a is abundantly expressed in rice seedlings and miR168b expression may be limited to specific cells or tissues or developmental stages that were not tested in this study. In rice, miR396 is represented by 3 variants with 5 loci. Previously, we reported the expression of only one variant which has 2 paralogous loci (miR396d, e) in rice [32]. Interestingly, the miR396d, e variant appears to be a monocot-specific version. Here we provide evidence for the expression of the other two variants although at lower levels compared to miR396d, e (Table 4). OsmiR396d, e appeared 20 times in the seedling libraries whereas the other two members i.e., miR396a, b and miR396c were represented only by 8 and 5 times respectively (Table 4). Similarly, miR164 has two variants coming from six loci in rice and only miR164c differs from the remaining five loci (miR164a, b, d, e, f). miR164c was represented by 12 reads and the other member by 55 reads. miR166 has 14 loci with 5 members and all were found to be expressed in rice but with varied frequencies. miR166i, j appeared only once whereas one other member appeared 55 times and the frequencies of the remaining members were between 1 and 55 reads (Table 4).

We also found cases in which all variants of a miRNA family were expressed at similar frequencies. For example, miR159 is represented by 3 family members and can be mapped to 6 loci in rice. All 3 members that differ in one nucleotide were found to be expressed at similar abundance (~90 reads). Similarly two other miRNAs, miR172 and miR167, are represented by 2 members each, and were expressed at a similar abundance (Table 4).

Some of the conserved miRNA family members were expressed at low levels in rice seedlings. miR162 is represented by two paralogous loci and expressed at a very low frequency. Similarly, miR319 and miR397 are represented by one member each but represented by two loci in the rice genome. These two miRNA families were found to be expressed at low levels. The lowest frequency was found in the case of miR394, miR395, 399 and 408, which were represented by only a few of reads in these libraries (Table 4). Thus far, miR394 has only been predicted based on sequence conservation with Arabidopsis [30] and it's expression is unknown in rice. Our sequence analysis indicated that miR394 is expressed in rice. These findings suggest that most of the members of conserved miRNA families are expressed. Furthermore, the expression profiles of different members/variants within a miRNA family can be similar or very different in rice seedlings, depending on the specific family. Among the known non-conserved rice miRNAs, miR820, miR535, miR818 and miR530 appeared 20 times or more, at least in one of the 3 libraries (Table 4). Thus our miRNA profiling in rice seedlings revealed the expression of 79 miRNA variants belonging to 46 miRNA families (Table 4).

### Expression analysis of new and candidate miRNAs

#### New miRNAs

**Figure 3:**
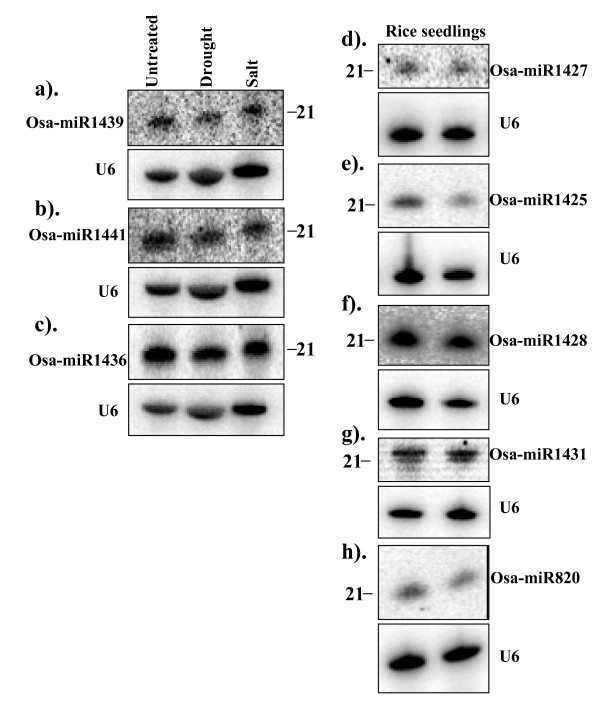
**Expression patterns of the new miRNAs in rice seedlings**. (a-c), Small RNA blots of low molecular weight RNA isolated from rice seedlings which were untreated (control) or treated with salt or drought stress. The blots were probed with ^32^P-end-labelled oligonucleotides; (d-h) Blots of low molecular weight RNA isolated from untreated rice seedlings in duplicates. The blots were stripped and re-probed with U6 as loading controls.

The variation in the frequencies of the new and putative new miRNAs among the 3 libraries may indicate possible regulation of these small RNAs under stress (Table 1 and Table 3). However, the observed differences for these non-conserved miRNAs are rather small and thus it is difficult to conclude whether or not these miRNAs are truly regulated by stress. To further characterize the expression (including stress regulation, if any) of some of the new as well as putative new miRNAs, we performed Northern blot analysis. Five of the new miRNAs (Osa-miR1425, Osa-miR1427, Osa-miR1428, Osa-miR1431 and Osa-miR1439) with single locus in rice genome could be detected using gel blot analysis out of eight miRNAs tested (Figure 3). We also detected another two miRNAs with more than one locus, i.e, Osa-miR1441 that has 3 loci and Osa-miR1436 that has 20 loci in the rice genome (Figure 3). The expression analysis also included Osa-miR820, a known miRNA in rice. Together, we were able to detect the expression of 7 of the new miRNAs, out of 10 examined (Figure 3). A comparison between sequence read frequency in the libraries and the Northern blot data revealed a rather complicated picture. For instance, Osa-miR1436 was represented by 77, 22 and 0 reads respectively in the control, drought and salt stress libraries (Table 1), indicating that it might be down-regulated, specifically under drought stress. However, the Northern analysis result is not in agreement with this assessment (Figure 3). Despite the fact that our drought library was represented by least number of rice genome matching small RNAs, Osa-miR1441 was represented by 24 reads in the drought stress library and 0 reads in the control library (Table 1) suggesting that this miRNA might be slightly induced under drought stress. However, Northern analysis showed that its expression levels did not differ between the control and stress conditions (Figure 3).

#### Candidate miRNAs

**Figure 4:**
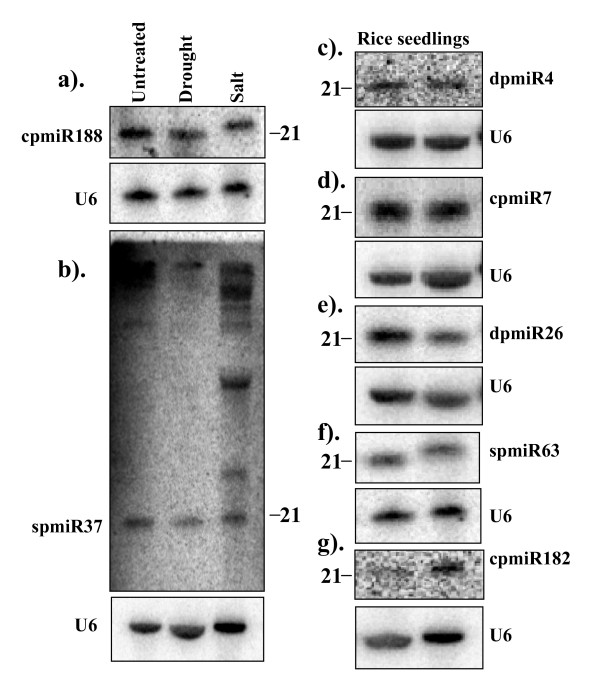
**Expression patterns of the newly identified putative miRNAs in rice seedlings**. (a-b), Small RNA blots of low molecular weight RNA isolated from rice seedlings which were untreated (control) or treated with salt or drought stress. The blots were probed with ^32^P-end-labelled oligonucleotides; (c-g) Blots of low molecular weight RNA isolated from untreated rice seedlings in duplicates. The blots were stripped and re-probed with U6 as loading controls.

Similarly, we also performed expression analysis of the candidate new miRNAs using small RNA blot analysis (Figure 4). Of the 13 putative new miRNAs tested, we were able to detect signals for 7 of them (spmiR63, dpmiR4, dpmiR26, cpmiR7, cpmiR182, cpmiR188, and spmiR37) (Figure 3). A candidate miRNA, cpmiR7 appeared 87 times in the control library and 0 times in the salt-stressed library suggesting that this might be suppressed under stress conditions, but no detectable differences were found in gel blot analyses (data not shown). Three other candidate miRNAs, dpmiR4, dpmiR26 and spmiR63, were represented by 10, 11 and 7 reads, respectively, in the stressed-libraries but none from the control library. Northern analysis indicated no differences between the control and stress treated seedlings (data not shown). Taken together, there did not seem to be a good correlation between the sequence reads data and Northern blot results. The apparent discrepancy may simply be due to the unreliability of the sequence reads data because of insufficient number of reads. It is also possible that the Northern analysis was not sensitive enough to reveal small differences. It has been suggested that cloning has greater sensitivity in terms of determination of miRNA abundance [26]. Notwithstanding this difficulty in determining the relative abundance of miRNAs, our detection of specific ~21 nt signals for some of the putative new miRNAs demonstrates that they are at least genuine small RNAs and not non-specific degradation products (Figure 4).

For most of the probes used in the Northern analysis, we noted that in addition to the signal of expected size, we detected signals of larger sizes specifically in the salt stress samples. One representative blot with prominent larger signals is shown in Figure 4b. The larger signals may correspond to miRNA processing intermediates, suggesting that salt stress may affect the processing of the small RNAs.

## Discussion

With the application of deep sequencing, we have identified mostly low-abundant new miRNAs [23] and putative new miRNAs [40] in rice. The identification of a large number of miRNAs not previously reported in rice, at least 6 of which are conserved in monocots, suggests that there may be many more monocot-specific or rice-specific miRNAs to be identified. It is evident that the identification of the complete miRNA repertoire in rice will require a broader sampling of tissues and deeper sequencing. It is known that the inflorescence expresses more diverse miRNAs in Arabidopsis [24-26]. Certainly, seedlings used in this study must be complemented with additional tissues such as inflorescence for further discovery of miRNAs in rice. This, coupled with similar studies in related monocots will help establish how many of these currently rice-specific miRNAs are conserved in other monocot species.

The number of deeply conserved miRNA families in rice largely remains the same as in Arabidopsis. However, rice appears to have evolved lineage-specific (monocot) miRNAs because of functional diversification between monocots and dicots. Using deep sequencing of small RNA libraries, we provided evidence for the expression of 79 miRNA variants belonging to 46 miRNA families as well as 23 new miRNAs and 40 putative new miRNAs. The nonconserved plant miRNAs presumably emerge and dissipate in short evolutionary time scales [25,26]. As proposed for *Arabidopsis *[25], high-throughput sequencing of small RNAs from species closely related to rice would help define the life span of the non-conserved miRNA genes.

The number of times each miRNA is represented in a small RNA library could serve as an index for the estimation of their relative abundance. The frequency of miRNA families in rice in our control library varied from 1 (miR394, miR399 and miR408) to 4948 times (miR169) indicating that the expression varies highly among different miRNA families in rice seedlings. The very high abundance of miR169 in rice seedlings is also in agreement with the data from a small RNA library generated using wheat seedlings [43].

Forty of the putative new miRNAs can each be mapped to between 1 to 306 loci (Table 3). At least some of these sequences could be potential miRNAs derived from novel repeat sequences that adopts hairpin structures. In animals, a large body of emerging evidence supports the view that some miRNAs are derived from repeat-rich regions. For instance, 10 mammalian miRNAs, including 6 human miRNAs have been shown to be derived from transposable elements [44]. In another report, it was confirmed that 50 human miRNAs reside within repetitive (Alu) elements [45]. Yet another recent report indicated that 75 miRNAs each in humans and mouse are likely derived from repeats [46]. Interestingly, mouse repeat-clustered miRNAs have more than 2000 genomic loci that are interspersed in the mouse genome. Furthermore, it was estimated that the miRNAs derived from repeats accounted for 23% of miRNA genes, although the cloning frequency was represented by only 2.6% of the total miRNA sequences [46]. A very low cloning frequency of these miRNAs suggests that they are low abundance miRNAs. Similarly, a human miRNA family (hsa-mir-548) was found to be derived from Made 1 elements (MITEs) [47]. Interestingly, Made 1 derived hsa-mir-548 miRNAs are generated from both strands of the transposable elements, as transcripts from both the strands were able to adopt hairpin like structures [47]. hsa-mir-548 family members can be mapped to between 20 to 145 loci in the human genome. Our observation that some of the putative miRNAs can be mapped to many loci with predicted fold-back structures raises the possibility that some of these could be authentic loci for miRNAs in rice.

Many conserved miRNA families target transcription factors but most non-conserved miRNAs likely target diverse genes that function in a broad range of biological processes. In an earlier study, we have reported that miR444 and it's target genes (2 MADS box factors) are conserved in monocots such as wheat, barley, maize, sorghum and sugarcane but not in *Arabidopsis *[32]. Identification of 5 new members (Osa-miR444c.1, c.2, d, e, f) that are highly homologous with miR444 indicated that miR444 and their conserved MADS box factor genes as targets of this miRNA family is interesting. These observations suggest that the 2 MADS box factors may have been regulated by different members of miR444 family in monocots. Furture studies focusing on functional analysis of these regulations by disrupting the miRNA-targeted site in these MADS box factors would reveal the role of these interactions in monocots. Interestingly, miR444 target sequences were found in MADS-box transcription factor genes in grape and soybean. miR444 does not seem to be present in soybean (Subramanian et al., unpublished data), it is not known whether or not miR444 homolog is present in grapes. These observations raises two possibilities; (1) monocots may have retained miR444 which regulates MADS box factors while dicots have lost after the divergence, (2) miR444 may have evolved in monocots after the divergence. Besides the MADS box factor, we predicted C3HC4 family protein as a target for ceratin members of this miRNA family.

Pentatricopeptide repeat genes form a large family and are implicated in post-transcriptional processes such as splicing, editing, processing and translation specifically in organelles such as mitochondria and chloroplasts [48]. Mutations in PPR genes result in a wide range of phenotypes including cytoplasmic male sterility [49,50]. In Arabidopsis, PPR transcripts are found to be targeted by miR400, miR161.1 and miR161.2 [51,52]. Recently, it was shown that miRNA targeted PPRs in *Arabidopsis *will give rise to phased siRNAs [52]. miR475 and miR476 in *Populus *are also targeting PPR transcripts [23]. The PPR targeting miRNAs in Arabidopsis and Populus are unrelated and this led to the hypothesis that miRNAs that target PPR genes appear to be independently evolved in Arabidopsis and rice. Similarly, we have predicted that Osa-miR1425 is targeting PPR transcripts in rice (Table 2) and Osa-miR1425 is not related to any of the miRNAs that are targeting PPR transcripts in *Arabidopsis *and *Populus *and raises the possibility that the Osa-miR1425 is a rice-specific miRNA that has evolved to suppress the PPR transcripts.

Four genes (3 of them encoding EF-hand proteins and one encoding a Calmodulin-binding protein) involved in calcium signaling are potential targets of 2 miRNAs (Osa-miR1432 and Osa-miR444d). Ca^2+ ^is a ubiquitous second messenger and triggers physiological changes in response to environmental stimuli [53]. The prediction that 4 components of Ca^2+ ^signal transduction are potential targets of 2 new miRNAs suggests a role for miRNAs in calcium signaling, one of the major signaling mechanisms implicated in diverse physiological processes in plants [53]. Identification of these predicted targets that are implicated in diverse biological processes expands the breadth of processes and pathways that are under miRNA regulation.

## Conclusion

The present study provides an important glimpse of small RNA abundance and diversity in rice. Our deep sequencing led to the identification of monocot-specific and rice-specific new miRNAs as well as candidate rice-specific miRNAs. Additionally, the expression of 79 miRNA variants belonging to 46 miRNA families was confirmed. Recently, Johnson et al., [38] and Nobuta et al., [39] found a large number of small RNAs in rice, suggesting extensive and complex regulatory roles of small RNAs in this plant. Our identification of new miRNAs and their predicted target genes adds to our understanding of the mechanisms regulating cellular function, and their evolution.

## Methods

### Cloning of rice small RNAs

Total RNA was isolated from the frozen seedlings by using the Trizol (Invitrogen, USA) according to the manufacturer's instructions. Cloning of the miRNAs was performed as described [51]. Briefly, low molecular weight RNA was enriched by NaCl and PEG precipitation. About 100 μg low molecular weight RNA was separated on a denaturing 15% polyacrylamide gel. Labeled RNA oligonucleotides with 18 and 26 nt were used as size standards. The nucleotides from 18 to 26 nt were excised, and RNA was eluted overnight with 0.4M NaCl at 4C. The RNA was dephosphorylated by alkaline phosphatase (Biolabs, New England) and recovered by ethanol precipitated. The small RNAs were then ligated sequentially to 5' (5'-tactaatacgactcactAAA-3'; uppercase, RNA; lowercase, DNA) and 3' (5'-pUUUaaccgcatccttctcx-3'; uppercase, RNA; lowercase, DNA; p, phosphate; x, inverted deoxythymidine) RNA/DNA chimeric oligonucleotide adapters. Reverse transcription was preformed after ligation with adapters, followed by PCR amplification. The resulting PCR products were sequenced by pyrosequencing method [54].

### Data analysis

The overall procedure of our computational methods for analyzing 454 small RNA libraries is shown in Additional file 3. In preprocessing (Additional file 3), all small RNA reads, which are referred to as reads thereafter, without perfect matches to the most proximal 11 nt of both adaptor sequences, which may be due to sequencing error, were first removed. Reads shorter than 17 nt were subsequently excluded from further analysis. Next, tandem and inverted repeats were removed using the Einverted and Etandem programs in the EMBOSS package [55], respectively. The repeat elements were filtered out with RepeatMasker [56] based on rice Repbase [57]. Unique reads mapped to rice tRNA, rRNA, snRNA, snoRNAs [58], known miRNAs [59] and TIGR Oryza Repeat Database [60] were removed. These pre-processing steps resulted in 58,781, 43,003 and 80,990 unique-genome matching small RNAs from the control, drought and salt stress libraries, respectively.

### Method for identifying new miRNAs

To deal with unique features of rice small RNA sequences, we developed a new method for identifying miRNAs from the 454 sequence libraries. Our method consists of several steps, as shown in Additional file 3. First, the reads that are shorter than 24 nt, and have more than one copy in a read library, fewer than 24 hits to the genome, and no match to known non-coding RNAs, were tested whether they are miRNAs, as follows. The lengths of 242 known rice miRNA precursors (pre-miRNAs) range from 60 to 312 nt, with a mean value of 145 nt. 217 out of the 242 (89.7%) known pre-miRNAs have no more than 200 nucleotides. In this work, we took 200-nt as the length of putative pre-miRNAs in our analysis. At each genomic locus of a short sequence to be tested, two sequences covering the read were extracted for secondary structure analysis, one sequence extending 160 nt upstream and 20 nt downstream from the read, and the other covering 20 nt upstream and 160 nt downstream of the read. The secondary structures for these two sequences were predicted by the RNA-fold program [61]. Those sequences that met the following two criteria were considered as candidate miRNA precursors. First, a secondary structure must have a hairpin with at least 18 paired nucleotides in its stem region. Second, the hairpin must have free energy less than or equal to -18 kCal/mol. and one central loop. In the biogenesis of miRNAs, other portions of pre-miRNAs will degrade soon after DCL1 separates miRNA:miRNA* duplex from the pre-miRNAs. Thus, the reads detected in the 454 libraries are supposed to be mature miRNAs or miRNA*. We thus checked the reads that are supposed to be mature miRNA or miRNA* to satisfy the requirements of the MIRCHECK program [31].

Next, the candidate precursor sequences were further tested by a two-class classification model. In this work, we adopted support vector machines (SVM) with a linear kernel [62] as the discriminative model. The SVM implementation that we used was from WEKA software package [62] with its default parameters. To build the classification model, 242 known rice pre-miRNAs in miRBase version 9 [63] were used as positive samples. These known pre-miRNAs have a mean length of 145 nt and a standard deviation of 55. For negative samples, we used rice coding sequences, which were downloaded from TIGR Rice Genome Annotation Database [64]. The chosen coding sequences were segmented, and the lengths of these segments follow a normal distribution with the mean value of 145 and standard deviation of 55, which were the same as those for the known pre-miRNAs. These segments were subsequently folded with RNAfold and filtered with the same criteria, i.e., greater than or equal to 18 paired nucleotides, folding energy not greater than -18 kCal/mol. and with one central loop. 242 segmented sequences satisfying these criteria were randomly chosen as the negative samples. Then, these 484 positive and negative samples were used to train a SVM model. The model used 4- to 9-mer short sequence motifs within the precursors as features. These sequence motifs were extracted by the WordSpy algorithm [65]. To improve classification accuracy, a wrapper-based feature selection method [66] was further applied to choose informative motif features. We adopted a 10-fold cross validation strategy. Both the positive samples and negative samples were randomly divided into 10 groups. One group of positive samples and one group of negative samples were combined to form a test set. Sequence motifs were extracted from the remaining 9 groups of positive samples. We first trained a SVM model using remaining 9 groups of positive samples and 9 groups of negative samples. The accuracy of the resulting SVM model was obtained by applying the model to the test set. To search for a good set of motifs (features), we then removed 5% of the motifs that have the least weights in the SVM model, as they provide the least amount of discriminative power. We then rebuilt a new SVM model using the remaining motifs, and tested its accuracy on the test set. We repeated this process and built a series of SVM models, until we had less than 20 motifs. Finally we took as the final set of features the motifs used by the SVM model that has the highest accuracy. We iterated the whole process 10 times. At each time, one group of positive samples and one group of negative samples were left as test samples. From each of these iterations, a set of motifs were obtained for the best SVM model in that iteration. Finally we combined these sets of best motifs and used them to build a discriminative model. The final model was applied to the candidate precursor sequences to predict novel pre-miRNAs.

### Target predictions

The known rice ORFs were downloaded from TIGR Rice Genome Annotation Database and used for target predictions for the putative miRNAs. We allowed only 3 mismatches between mRNA target and putative new miRNA in our predictions [40].

### RNA gel blot analysis

Total RNA was also isolated from four-week-old rice seedlings either untreated (control) or exposed to and salt stress or drought stress using Trizol Reagent. Low molecular weight RNA was isolated from total RNA using PEG precipitation. Twenty microgram low molecular weight RNA was loaded per lane, resolved on a denaturing 15% polyacrylamide gel, and transferred electrophoretically to Hybond-N+ membranes. Membranes were UV cross-linked and baked for 2 hours at 80°C. DNA oligonucleotides complementary to miRNA sequences were end labeled with γ-^32^P-ATP using T4 polynucleotide kinase (New England Biolabs). Membranes were prehybridized for at least 1 hour and hybridized overnight using Perefect hybridization buffer (Sigma) at 38°C. Blots were washed three times (two times with 2 × SSC + 1% SDS and one time with 1 × SSC + 0.5% SDS) at 50°C. The membranes were briefly air dried and then exposed to phosphorscreen and images were acquired by scanning the films with a Typhoon.

## Authors' contributions

R.S. and J.-K.Z. designed the research. R.S. constructed the small RNA libraries and performed the expression analysis. XZ, YZ and WZ designed the computational methods, analyzed the data and results and wrote the sections and parts on computational methods and results. XZ and YZ developed the software and performed the experiments. R.S. wrote the paper; J.-K.Z. edited the paper. All authors read and approved the final manuscript.

## Supplementary Material

Additional file 1 Predicted fold-back structures using precursor sequences of newly identified miRNAs in rice. Predicted fold-back structures using precursor sequences of newly identified miRNAs in rice. Click here for file

Additional file 2 Predicted targets for the newly identified putative miRNAs in rice. Predicted targets for the newly identified putative miRNAs in rice Click here for file

Additional file 3 Schematic representation of the procedure used to identify new miRNAs in rice. Schematic representation of the procedure used to identify new miRNAs in rice Click here for file
