# Supplementary material for: Identification of novel and candidate miRNAs in rice by high throughput sequencing
Source: BMC Plant Biol. 2008 Feb 29;8:25. doi: 10.1186/1471-2229-8-25 (PMC2292181; doi:10.1186/1471-2229-8-25)
Supplement: Additional file 1 — Predicted fold-back structures using precursor sequences of newly identified miRNAs in rice. Predicted fold-back structures using precursor sequences of newly identified miRNAs in rice. [file 1471-2229-8-25-S1.doc]

**Osa-miR167***

**- u a c - uc a cg ug-- uac g**

**gugugaa gaguga gcug ca gcaugaucuagc ugauu au gcac uuggcg aguc a**

**cacacuu UUUACU CGAC GU CGUACUAgauug acuaa ug ugug gacugc ucag u**

**u c C A A uc a ug ucua uaa u**

**Osa-miR810.b2**

**aaua c cu**

**uguggcucgcaugcuua acuaacggcauaauuagaucacuugauga gacguauaucgguguucgcuauauauacuaucuacugguaaguauau \**

**acaccgagcguacgaau ugaUUGCCGUAUUAAUUUAGUGAAcuacu cugcguauagccacaagugauauauaugauagaugaccauucauaua u**

**---- a au**

**Osa-miR1423**

**cc--- a a u a - a - c-| g**

**cau gcaca cac aguauuugggaggcaacuac cg uugggcgcucgaucc ggggu gggaaaau gg c**

**gua cgugu gug ucauaaacccucuguugaug gc aacccgcgagcuagg cccca cccuuuug cc g**

**cguac - c u - g c c ca^ c**

**Osa-miR1425**

**uugacugc auc a ---| uau**

**auuaggauuca cuugcugcu aau guauugcu \**

**uaauucuaggu gaacgacga uug uauaacga a**

**ua------ caa c uaa^ cuu**

**Osa-miR1427**

**a a agc uc c c a g a c cguua .-ccaacug| ug**

**--gaccg uucguug ugcgg ggaugcgugggaccgccgguccu ggcuuggcugcgcgcgac gcgc gucccgcaggcc gcgccgcgccaccgcgcgguuccgcagca gg caugcgc acc cag aaauc c**

**cuggc aagcagc gcgcc ccuacgcacccuggcggccaggg ccgaaccgacgcgcgcug cgcg cagggcguccgg cgcggcgcgguggcgugccaaggcgucgu cc gugcgcg ugg guc uuuag a**

**c c gua ca a c c g g c cuuag \ -------^ uc**

**Osa-miR1428**

**agu a cc ugaa g au u**

**gcguuuugcaaauucgc ggc uaucuuguggua cu aguacgcg ga u**

**ugcaaaacguuuaagug ccg auagaauaccau gg uuaugcgu cu u**

**--- - aa ucgg g c- a**

**Osa-miR1429**

**ACCCAGAUGAAG U U U UU------------ U - GUG**

**GUAAUAUAC AAUCCGUGCA CCA CGUAUAUC ACACG CAU GUGAAC C**

**CGUUGUAUG UUGGGCACGU GGU GUGUAUGG UGUGU GUA UACUUG A**

**------------ U U - UAAUUGUAAAUAUU U A GCU**

**Osa-miR1430**

**gu- --- ua a- gauugc g a u ug u - c g - - acac**

**gaggaggg uagccu caugg gaugagga cucu uuagcca gaa ggcu cc aucu cca uauuu guucauca cug gaac \**

**uuucuccc gucgga guacc cuacucuu gaga aaucggu cuu ccga gg uaga ggu auaaa uagguggu gac cuug u**

**auc uau uc cc agu--- g c - gu - a - g a u gggu**

**Osa-miR1431**

**uucagaaagga ----- u c ug cg ggc u a .-auaaaugaguuua a ---- - ca**

**aagac uuaggg ugcaagcgggucaac cg aacc cuuaua aaaau agu gguaacccgu ugg ucga cc cacuug u**

**uucug gauccc acguucgcccgguug gc uugg gaauau uuuua uca cuauugggca acc ggcu gg gugaau c**

**a---------- ccgua u a gu au aca u - \ ------------- a aaac c cu**

**Osa-miR1432**

**agg- a ug uug ggu- ug gc a a ga c- ------ cu cu**

**gagg ug cguuc ugggg u g ccugug ucagg gagaugacacc caucg cgga auucguu uggu \**

**cucc au guagg guccc a c ggauac agucc cucuacugugg guagu gccu uaaguag accg u**

**guag - gu uaa aguc gu a- a c ac uu gguagu u- ug**

**Osa-miR1435**

**C U uu a .-aaaa| a**

**acuUUU UUAAGUCAAACU UUUu ag uuugaucaaauuuau aaaauau g**

**ugaaaa aauucaguuuga gaag uc aaauuaguuuaaaua uuuuaua c**

**a u uu - \ ----^ a**

**Osa-miR1436**

**-------- a c**

**aguuuaugu gcuacucccuccgucccauaau uuaaaaccuaggacuggauaggacguuucauaguacaaugacu \**

**ucaaauaua ugaUGAGGGAGGCAGGGUAUUA Aauuuuggauccugaccuauccugcaaaguaucauguuacuga a**

**uccaacuc C g**

**Osa-miR1437**

**c a a gugaggug ac ca gc a u**

**gcgcg ggggagggagggaacggugccuagugcggc ccggagcucgccagcacgc gga aggagg ggcggc gg gcggag gcu g**

**cgcgc ccccucccuccuuuGUCACGGAUCACGCCG GGCCUcgagcggucgugcg ccu uccucc ucgccg cc cgccuc cgg c**

**a C - -------- a- -- ac g c**

**Osa-miR1438**

**uga aag u C c c a c cuauguaaa ua- u**

**guug uau gu uuAGGGUAAUUUUAU AUUUUUAAgaaaacaaaauuuaauac uu agau uu gguau aaauuuu ga c**

**uaac aua ca aaucccauuaaaaua uaaaaauucuuuuguuuuaaauuaug aa ucua aa ccaua uuuaaaa cu c**

**ucc aua u u c a a u a-------- uaa c**

**Osa-miR1439**

**aauc ug uuug g ac c c c cua u-- cua**

**uau u uuauaaau uaauacu cuccgu ccaaaauauag cauuuuuag uaugaau gacauugu uuuag u**

**aua g gauguuua aUUAUGA GAGGCA GGUUUUauauu guaaaaauc auacuua cuguaaca agauc g**

**---- gu uuuu a GU A c a aac uac uaa**

**Osa-miR1440**

**a c c c .-uc ag**

**aaaugcca UGCUCAAAUACCACUCUCCUaaauuu cauu ccaaauacca ccgggcccacaugucagcc aucc c**

**uuuacggu acgaguuuauggugagaggguuuaaa guaa gguuuauggu ggcccggguguacagucgg uggg a**

**c a u a \ -- ac**

**Osa-miR1441**

**a c c cc c a gg**

**guuuuuu auucguguc gaaaacu uuuugauaucuggu aaac uucgaugugacaucuaaaaauuuucuuuucgcgaacuaag c**

**caaaaaa uaagcacag cuUUUGG AAGGCUGUAGGCCA uuug aggcuacacuguggguuuuuaaaagaaaagcgcuugauuu c**

**- a a AA a c gu**

**Osa-miR1442**

**aua u AG - a gua g- gg- u aag a---- cuaagug cu**

**ucua AUUCAU UACUAG AUGUGUc caucca cua guu ugu uuaugggacg ggaguag ucuuuug gagg a**

**aggu uaagua augauc uacacag guaggu ggu uag aua aauacccugc ccucauc aggagac cucu a**

**--- c aa c c g-- aa gga u cua auaug uacua-- ua**
