# Supplementary figures and images for: Identification of novel and candidate miRNAs in rice by high throughput sequencing
Source: BMC Plant Biol. 2008 Feb 29;8:25. doi: 10.1186/1471-2229-8-25 (PMC2292181; doi:10.1186/1471-2229-8-25)

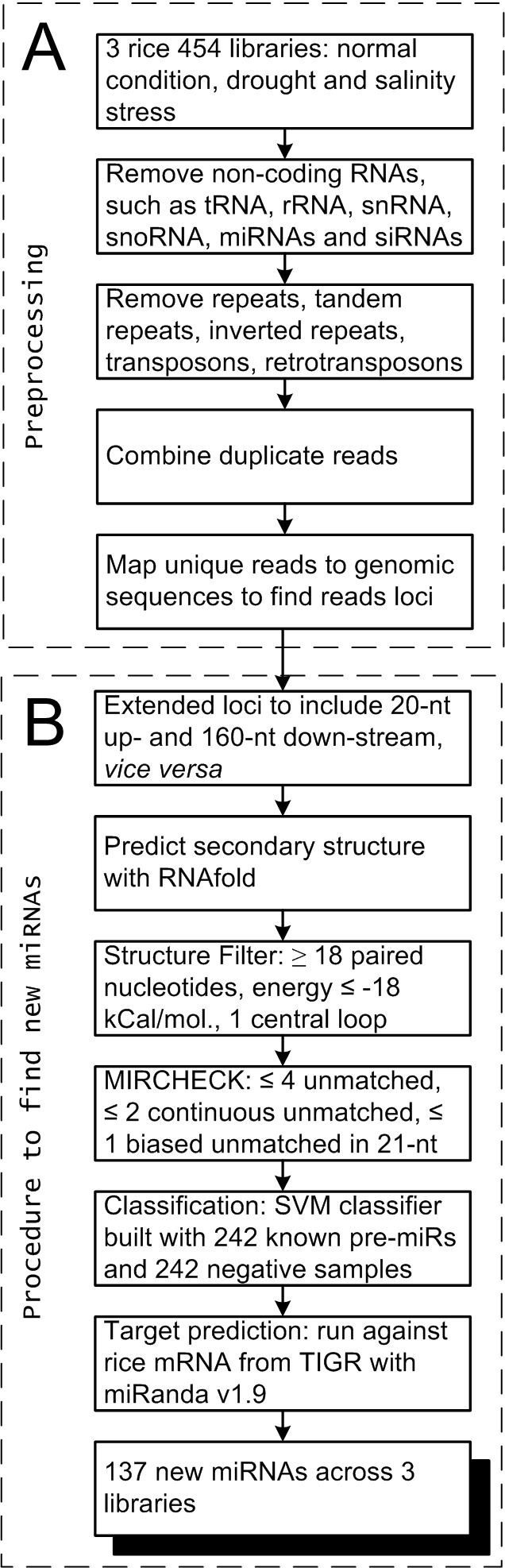

Supplement: Additional file 3 — Schematic representation of the procedure used to identify new miRNAs in rice. Schematic representation of the procedure used to identify new miRNAs in rice [file 1471-2229-8-25-S3.jpeg]
